# Genomic surveillance of SARS-CoV-2 in COVID-19 vaccinated healthcare workers in Lebanon

**DOI:** 10.1186/s12920-023-01443-9

**Published:** 2023-01-27

**Authors:** Habib AlKalamouni, Farouk F. Abou Hassan, Mirna Bou Hamdan, Andrew J. Page, Martin Lott, Michaela Matthews, Nada Ghosn, Alissar Rady, Rami Mahfouz, George F. Araj, Ghassan Dbaibo, Hassan Zaraket, Nada M. Melhem, Ghassan M. Matar

**Affiliations:** 1 Department of Experimental Pathology, Immunology, and Microbiology, Center for Infectious Diseases Research, Faculty of Medicine, American University of Beirut, Beirut, 1107 2020 Lebanon; 2 Center for Infectious Diseases Research, Faculty of Medicine, American University of Beirut, Beirut, Lebanon; 3 Medical Laboratory Sciences Program, Division of Health Professions, Faculty of Health Sciences, American University of Beirut, Beirut, 1107 2020 Lebanon; 4 Quadram Institute Bioscience, Norwich Research Park, Norwich, UK; 5 Epidemiological Surveillance Unit, Ministry of Public Health, Beirut, Lebanon; 6 World Health Organization, Beirut, Lebanon; 7 Department of Pathology and Laboratory Medicine, Faculty of Medicine, American University of Beirut, Beirut, Lebanon; 8 Department of Pediatrics and Adolescent Medicine, Faculty of Medicine, American University of Beirut, Beirut, Lebanon

**Keywords:** SARS-CoV-2, Variants of concern, Genomic surveillance, Healthcare workers, NextSeq 500, Lebanon

## Abstract

**Background:**

The emergence of SARS-CoV-2 variants including the Delta and Omicron along with waning of vaccine-induced immunity over time contributed to increased rates of breakthrough infection specifically among healthcare workers (HCWs). SARS-CoV-2 genomic surveillance is an important tool for timely detection and characterization of circulating variants as well as monitoring the emergence of new strains. Our study is the first national SARS-CoV-2 genomic surveillance among HCWs in Lebanon.

**Methods:**

We collected 250 nasopharyngeal swabs from HCWs across Lebanon between December 2021 and January 2022. Data on the date of positive PCR, vaccination status, specific occupation, and hospitalization status of participants were collected. Extracted viral RNA from nasopharyngeal swabs was converted to cDNA, library prepped using the coronaHIT method, followed by whole genome sequencing on the Illumina NextSeq 500 platform.

**Results:**

A total of 133 (57.1%) samples belonging to the Omicron (BA.1.1) sub-lineage were identified, as well as 44 (18.9%) samples belonging to the BA.1 sub-lineage, 28 (12%) belonging to the BA.2 sub-lineage, and only 15 (6.6%) samples belonging to the Delta variant sub-lineage B.1.617.2. These results show that Lebanon followed the global trend in terms of circulating SARS-CoV-2 variants with Delta rapidly replaced by the Omicron variant.

**Conclusion:**

This study underscores the importance of continuous genomic surveillance programs in Lebanon for the timely detection and characterization of circulating variants. The latter is critical to guide public health policy making and to timely implement public health interventions.

**Supplementary Information:**

The online version contains supplementary material available at 10.1186/s12920-023-01443-9.

## Background

Since its emergence in December 2019, severe acute respiratory syndrome coronavirus 2 (SARS-CoV-2) remains a global public health threat. According to the World Health Organization (WHO), excess mortality attributed to COVID-19 had reached 14.9 million between January 2020 and December 2021, with middle-income countries accounting for 81% of the death toll [1]. In Lebanon, more than 1.1 million confirmed cases and 10,457 deaths have been reported [2]. Vaccine development against SARS-CoV-2 proceeded in an unprecedented pace with 11 vaccines granted emergency use listing (EUL) by the World Health Organization (WHO) as of July 25, 2022 [3]. More than 11.4 billion vaccine doses have been administered worldwide since the start of COVID-19 vaccine rollout in December 2020 [4]. Despite the availability of effective vaccines against SARS-CoV-2, according to a modeling study, it is generally accepted that vaccine-induced immunity wanes overtime leading to an increased incidence of breakthrough infections, especially with the emergence of new variants [5].

Since the emergence of SARS-CoV-2, new variants have evolved from the original SARS-CoV-2 strain (Wuhan 19 strain (WA1/2020)). These variants are classified into variants under monitoring (VUM), variants of interest (VOI), and variants of concern (VOC) [6, 7]. VUM are variants with genetic changes that are suspected to affect virus characteristics and may pose future risk but with yet no clear evidence of phenotypic or epidemiological impact. VOI are SARS-CoV-2 variants possessing predicted or known genetics changes that affect the characteristics of the virus (transmissibility, disease severity, immune escape, therapeutic escape) and known to cause significant community transmission or multiple COVID-19 clusters in multiple countries. VOC are SARS-CoV-2 variants that meet the definition of a VOI and are associated with increased transmissibility detrimental change in COVID-19 epidemiology, increased virulence, changed clinical disease presentation, and decreased effectiveness of public health and social measures, vaccines, or therapeutics against the virus. To date, the WHO has identified five VOCs worldwide: Alpha (B.1.1.7 lineage) first detected in the United Kingdom (UK), Beta (B.1.351 lineage) first detected in South Africa, Gamma (P.1 lineage) first detected in Brazil, Delta (B.1.617.2 lineage) first detected in India and Omicron (B.1.1.529 lineage) first reported in South Africa [7]. Moreover, several VOI have been identified in several countries; these include B.1.427 and B.1.429 from the USA (California, WHO alert since July 6, 2021), B.1.525 from the UK  and Nigeria, B.1.526 from the USA, B.1.617.1 and B.1.617.3 from India, P2 from Brazil, and C.37 from Peru [6]. The WHO is continuously monitoring and assessing the evolution of SARS-CoV-2 and the emergence of new variants with increased risk to global public health.

The B.1.617.2 lineage along with its sub-lineages made up the Delta variant that was responsible for the COVID-19 surge in India, eventually spreading and dominating globally [8]. Despite the high replicative efficiency, reduced sensitivity to host immune responses, and high transmissibility of the Delta variant compared to previous VOCs, vaccine effectiveness was sustained [8]. In November 2021, the surge of cases in South Africa marked the identification of a new VOC named Omicron (B.1.1529). Omicron replaced the Delta variant and was characterized by a higher number of amino acid substitutions, higher transmissibility and partial resistance to vaccine induced immunity compared to previous VOCs [9–11]. Studies showed that although Omicron had higher rates of reinfection, it was clinically less severe compared to the Delta variant, possibly due to prior infections and T cell mediated immune responses [8]. Recent studies showed that vaccine effectiveness against the Omicron variant (B.1.1.529) was lower than the Delta variant (B.1.617.2) after primary immunization with 2 doses of the ChAdOx1 nCoV-19, BNT162b2 or mRNA-1273 vaccines with significant reduction in vaccine effectiveness against the two variants ≥ 25 weeks following the second dose [12]. Nevertheless, vaccine effectiveness against symptomatic disease was restored following a booster shot, underscoring the importance of a third dose [13, 14].

In Lebanon, 43.6% of the population were fully vaccinated (2 doses) as of June 2022 [2]. Healthcare workers (HCWs) are at increased risk of SARS-CoV-2 infection compared to the general population and have been prioritized in COVID-19 vaccine rollout worldwide [15] as per the WHO Strategic Advisory Group of Experts framework for the allocation and prioritization of COVID-19 vaccination [16] and the Advisory Committee on Immunization Practices [17]. Consequently, HCWs were among the priority groups to receive the BNT162b2 vaccine in Lebanon. Vaccine rollouts started in mid-February 2021 and the second dose was administered 21 days following the first dose. In October 2021, the third dose (i.e. booster shot) was introduced [18]. The use of personal protective equipment (PPE) including mask mandates (for patients and visitors of healthcare facilities) significantly reduced the occupational risk of acquiring COVID-19 by HCWs [19]. However, waning immunity and the emergence of antigenically drifted VOCs meant that HCWs are at risk of breakthrough infections [20–27].

Genomic surveillance of SARS-CoV-2 was first initiated in Lebanon in 2020 with direct support from the WHO [28]. Lebanon was the first in the Eastern Mediterranean Region (EMR) to identify the Delta variant in a timely manner. However, the sampling was done randomly and mainly on travelers. In collaboration with the Ministry of Public Health (MoPH) and support from the WHO, this study allowed for the establishment of the capacity to perform an ongoing genomic surveillance of SARS-CoV-2 among HCW’s to support the timely implementation of public health measures to control the spread and emergence of new SARS-CoV-2 variants and to inform global surveillance. Here, we report the first genomic data from this surveillance effort focusing primarily on breakthrough infections detected among HCWs, a group with the highest vaccination coverage among the Lebanese population.

## Methods

### Study design, population and data collection

This study is part of a national surveillance program in collaboration with the Epidemiological Surveillance Unit (ESU) at the Lebanese MoPH. Accordingly, a waiver of informed consent was granted by the Institutional Review Board (IRB) at the American University of Beirut (AUB). Between December 1, 2021 and January 31, 2022, nasopharyngeal swabs were collected from a total of 250 COVID-19-positive HCWs from five Lebanese healthcare centers. Samples with *Ct values* of less or equal to 25 were used. The majority of samples (n = 205) were collected from three hospitals in Beirut: AUB Medical Center (n = 175), Rasoul Al Aazam Hospital (n = 25) and Belle Vue Hospital (n = 5). The remaining were collected from Hammoud Hospital in South Lebanon (n = 26) and Mount Lebanon Hospital in Mount Lebanon (n = 19). Aliquots of the collected samples were stored at − 80 °C until processed. The date of positive PCR, vaccination status, specific occupation, and hospitalization status of participants were collected.

### RNA extraction and whole genome sequencing (WGS)

Aliquots of the nasopharyngeal swabs (140 µl) were used to extract total RNA following manufacturer’s instructions (QIAamp Viral RNA mini Kit, QIAGEN, Hilden, Germany, Cat. 52906). Aliquots were eluted in 30 µL of Buffer AVE. Both the concentration and quality of RNA samples were measured and checked with the Denovix Blue DS-11 Spectrophotometer. Viral RNA extracts were sequenced at the Quadram Institute Bioscience, UK. Briefly, viral RNA was converted to cDNA then amplified using the ARTIC protocol v3 (LoCost) [29] and using V4 of the primer set, with sequencing libraries prepared using CoronaHiT as previously described [30]. Genome sequencing was performed using the Illumina NextSeq 500 platform (Illumina, CA, USA) with one positive control and one negative control per 94 samples. The raw reads were demultiplexed using bcl2fastq (v2.20). The reads were used to generate a consensus sequence using the ARTIC bioinformatic pipeline [31]. Briefly, the reads had adapters trimmed with TrimGalore [32] and were aligned to the WuhanHu-1 reference genome (accession MN908947.3) using BWA-MEM (v0.7.17) [33]. The ARTIC amplicons were trimmed and a consensus was built using iVAR (v.1.3.1) [34]. PANGO lineages were assigned using Pangolin (v3.1.20) [35] and PangoLEARN model dated 2022-02-02 [36]. In this manuscript, we used the Pango lineage designation system.

## Results

### Demographic characteristics of study participants

Our study included a total of 250 HCWs testing positive for SARS-CoV-2 by RT-PCR between December 1, 2021 (n = 27), and January 31, 2022 (n = 223). Data on the occupation and COVID-19 vaccine status were available for only 175 HCWs working at AUBMC. Among those, the majority were females (64%) and received at least two doses of the BNT162b2 COVID-19 vaccine (95.4%). Nurses (39.4%), medical doctors (14.8%) and technicians (10.3%) accounted for the majority of samples (Table 1). None of the SARS-CoV-2-positive HCWs were hospitalized.

**Table 1:** Demographic characteristics of HCWs recruited at AUBMC

Variable	N	%
*Gender*		
Males	63	36
Females	112	64
*Vaccination status*		
≥ 2 doses	167	95.4
1 dose	4	2.3
Unvaccinated	1	0.6
Not documented	3	1.7
*Occupation*		
Nurses	69	39.4
Medical doctors	26	14.8
Technicians	18	10.3
Clerks, tellers and clinic assistants	16	9.1
Pharmacists	5	2.8
Physical therapists	2	1.1
Others^a^	40	22.8

^a^Administrative staff, research assistants, janitors and security guards

### SARS-CoV-2 phylogenetic analysis

**Fig. 1:**
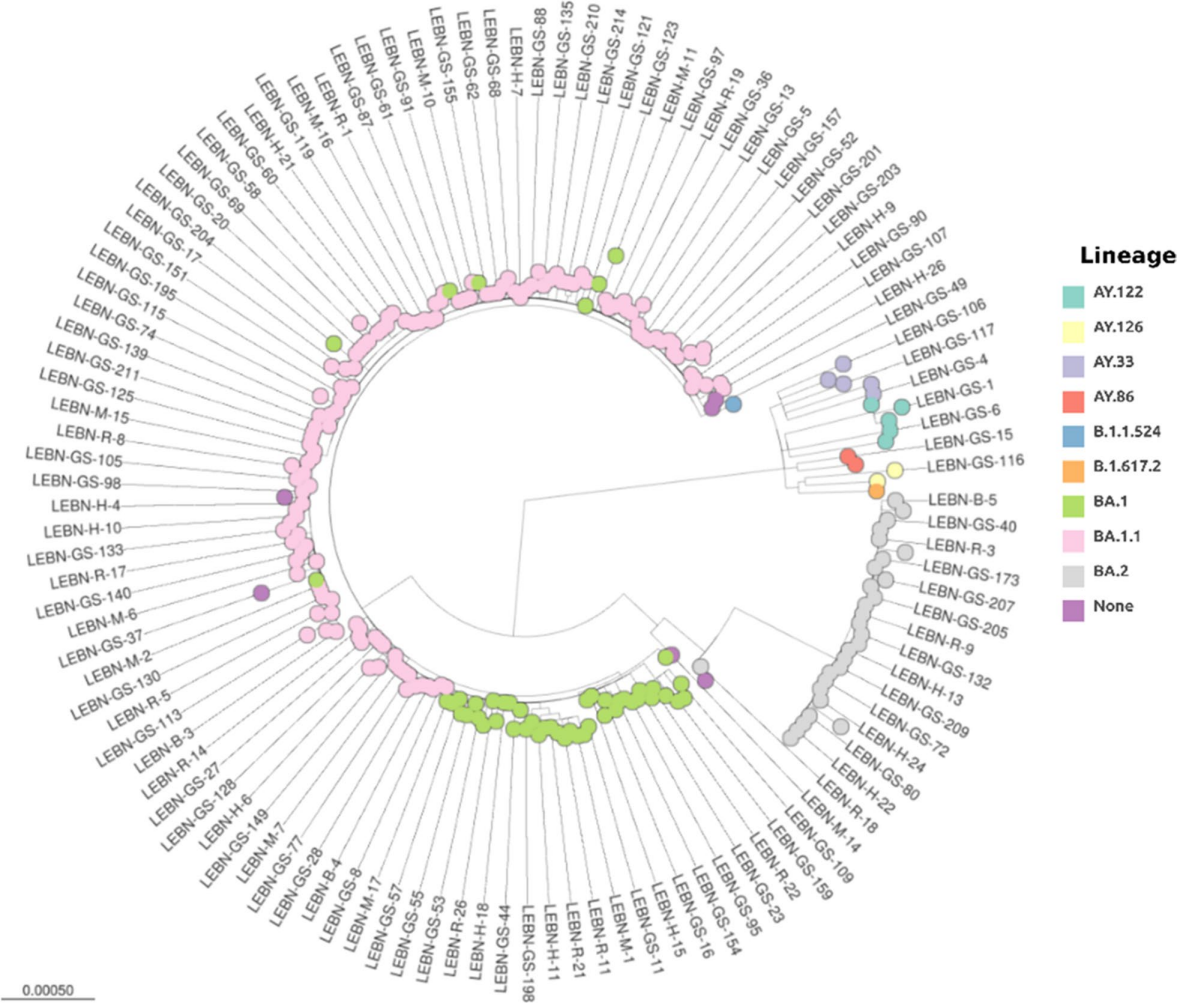
Phylogenetic analysis. Phylogenetic analysis of SARS-CoV-2 genome in 250 samples collected from HCWs in Lebanon. Each lineage is specified with a unique color

**Fig. 2:**
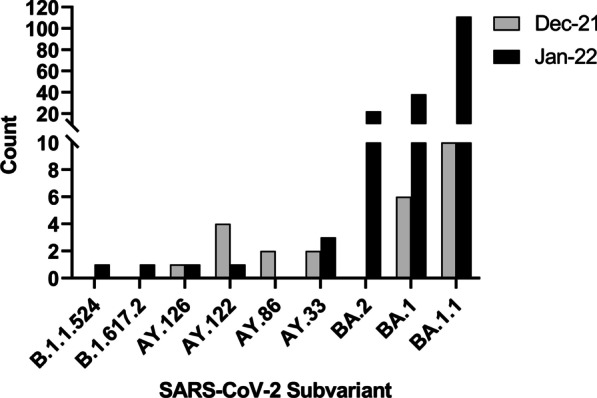
Frequency of SARS-CoV-2 lineages among HCWs. Data present the number of samples with a specific SARS-CoV-2 lineage detected in December 2021 (n = 27) and January 2022 (n = 198) out of 225 samples with available date of PCR testing

Lineage analysis was performed using the Pangolin COVID-19 lineage assigner. Overall, 10 lineages were identified among HCWs (Table 2; Additional file 1: Fig. S1). A total of 17 samples did not yield sufficient sequencing data to provide a lineage. This was most likely due to sample storage and handling errors as they passed through multiple labs in multiple countries. Consequently, we excluded them from the analysis. As expected, the Omicron variant was the predominant VOC (90.6%) detected in most of our analyzed samples followed by the Delta variant (6.4%). The predominant lineages identified were BA.1.1 and BA.1 accounting for 57.1% and 18.9% of the samples, respectively, followed by BA.2 (12%) (Table 2; Fig. 1). Sporadic detection of other lineages such as Delta was also observed. The collection date was available for a total of 225 samples. Out of those, 27 were collected in December 2021 and 198 were collected in January 2022. Our results revealed that Omicron BA.1.1 variant was the predominant VOC circulating in December 2021 (37%) and January 2022 (56%) followed by 22.2% and 19.2% BA.1, respectively (Fig. 2). Omicron BA.2 variant was detected in January in 22 (9.8%) of our samples whereby the majority (68.2%) were collected post January 20, 2022 (data not shown).

**Table 2:** SARS-CoV-2 lineages and variants detected in HCWs

Lineage	Variant	N	%
BA.1.1	Omicron (BA.1-like)	133	57.1
BA.1	Omicron (BA.1-like)	44	18.9
BA.1	Probable Omicron (BA.1-like)	5	2.1
BA.2	Omicron (BA.2-like)	28	12
BA.2	Probable Omicron (BA.2-like)	1	0.4
AY.33	Delta (B.1.617.2-like)	5	2.1
AY.86	Delta (B.1.617.2-like)	2	0.9
AY.122	Delta (B.1.617.2-like)	5	2.1
AY.126	Delta (B.1.617.2-like)	2	0.9
B.1.617.2	Delta (B.1.617.2-like)	1	0.4
B.1.1.524	Not applicable	1	0.4
None	Omicron (unassigned)	2	0.9
None	Probable Omicron (unassigned)	4	1.7
Total		233	100

## Discussion

Whole genome sequencing is important to characterize circulating SARS-CoV-2 variants and to detect emerging variants. In Lebanon, data on SARS-CoV-2 genome diversity and circulating lineages are limited specifically among HCWs who are at high risk of acquiring the infection. Genomic analysis of 11 specimens collected early during the pandemic in Lebanon (February– March, 2020) showed that the B.1 lineage was the most prominent, followed by the B.4 lineage and the B.1.1 lineage [37]. Between February 2020 and January 2021, the most frequently reported SARS-CoV-2 lineage among 58 analyzed samples was B.1.398 followed by B.1.1.7 and B.1 [38]. Moreover, an analysis of 905 samples showed the rapid emergence and dominance of the B.1.1.7 Alpha variant between January and April 2021 followed by the replacement of the Alpha variant with Delta variant between June and July [28]. Our study reveals that Omicron BA.1.1 followed by BA.1 and BA.2 predominated during January 2022 with the frequency of BA.2 gradually increasing by the third week of the latter.

The Omicron variant (B1.1.529) was first detected in early November 2021 in multiple countries and has been designated as a VOC by the WHO on November 26, 2021 [39]. Four Omicron lineages were first identified: B1.1.529, BA.1, BA.2, and BA.3 [40]. Recently, four novel Omicron subvariants designated as BA.4, BA.5, BA.2.12.2 and BA.2.13 have also emerged and started spreading globally [41–44]. Compared to BA.2, these novel omicron subvariants exhibit additional mutations in the spike region namely L452Q for BA.2.12.1, L452M for BA.2.13, and L452R + F486V for BA.4 and BA.5 [41]. These mutations have been shown to provide potential immune escape characteristics and higher transmission than BA.2 [41, 44]. In this study, we found that Omicron BA.1.1 and BA.1 lineages were the predominant circulating lineages in our cohort of HCWs between December 2021 and January 2022. The Delta variant was detected in only 6% of our samples suggesting the replacement of the Delta variant with Omicron as the predominant circulating VOC which is consistent with global trends observed during the same period [8]. We did not detect BA.3 lineage or any recombinant lineages in our sequenced samples. The former does not have specific mutations in the spike protein but rather a combination of mutations from BA.1 and BA.2 [45]. The rate of spread of the three Omicron lineages (BA.1, BA.2 and BA.3) differs with BA.1 and BA.2 being the predominant lineages. Between December 2021 and January 2022, BA.1 lineage accounted for 78% of sequenced samples submitted to the GISAID database compared to 16% of BA.2 [46]. This is similar to our findings reflecting the predominance of BA.1 over BA.2.

The subvariant BA.2 shares 32 mutations with BA.1 but has 28 distinct mutations, four unique ones in the RBD region alone, which according to a deep learning algorithm, made it far more likely than other lineages to be the next dominant subvariant [40]. Indeed, BA.2 had already become the dominant variant in multiple countries such as Denmark and UK in February 2022 [47]. Moreover, as of June 21, 2022, 52% of sequenced samples submitted to GISAID database were BA.2 followed by 19% BA.2.12.1, 18% BA.5, 11% BA.4 and < 1% BA.1 [46]. As we continue our national genomic surveillance beyond January 2022, we expect a shift in dominance in favor of the highly transmissible BA.2 subvariant as well as an expected detection of other Omicron subvariants.

Following the Delta variant wave in August 2021, the number of confirmed cases in Lebanon was relatively stable with an average of 4000 cases reported weekly. On December 22, the number of cases doubled with a weekly change rate of 69.51%, marking the beginning of a new wave of SARS-CoV-2. The numbers kept increasing gradually reaching a peak of 57,800 weekly cases on January 31. All the samples included in our study were collected during this period, and 88% of them belonged to the Omicron variant. According to the WHO COVID-19 weekly update, a similar trend was observed globally during the week of 27 December until January 2, where the total number of new cases exhibited a sharp increase of 71% as compared to previous weeks. In addition, GISAID variant tracking revealed a predominant global circulation of the Omicron variants in more than 75 countries. Data on vaccines effectiveness and breakthrough infections against SARS-CoV-2 variants are lacking in the Eastern Mediterranean region. However, a study conducted in Qatar predicted the cumulative incidence of breakthrough infections to be 0.59% and 0.84% 6 months following receiving the second dose of the mRNA-1273 vaccine and the BNT162b2 vaccine, respectively [48].

A key limitation of our study is that it did not include samples from all regions in Lebanon and thus is not fully representative of the situation in Lebanon. However, given that Beirut is the capital of this small country with a lot of population movement during the week, and particularly on the weekends when its residents travel to their villages across Lebanon, we believe that data from Beirut are to some extent representative of the whole country. We were also unable to gather clinical data of HCWs, which hampered our ability to assess risk factors associated with Omicron breakthrough infections. Moreover, data on receiving the date of the second and booster shots were unavailable and thus we were unable to estimate vaccine effectiveness between the date of receiving the booster and the date of breakthrough infection.

## Conclusion

Our findings underscore the importance of continuing genomic surveillance in Lebanon in order to monitor virus evolution and the emergence of novel SARS-CoV-2 variants. This is particularly important in HCWs as they are more likely to be exposed to emerging variants and can act as an advanced warning proxy to the wider community. More recently, two Omicron lineages (BA.4 and BA.5) have been identified in South Africa before being detected in several countries worldwide including Botswana, Belgium, Denmark, the United Kingdom, France, Germany, Portugal and China [42, 49, 50]. Therefore, continuing genomic surveillance will help assessing the characteristics and the public health implications of these lineages and other variants that might emerge and contribute to more informed public health intervention strategies.

## Supplementary Information

**Additional file 1. Fig. S1**: SARS-CoV-2 lineages and variants detected among the sequenced samples.

## Data Availability

The dataset(s) supporting the conclusions of this article is (are) included within the article itself.

## Abbreviations

HCWs: Healthcare workers
SARS-CoV-2: Severe acute respiratory syndrome coronavirus 2
COVID-19: Coronavirus disease 2019
EUL: Emergency use listing
WHO: World Health Organization
MoPH: Ministry of Public Health
ESU: Epidemiological Surveillance Unit
AUB: American University of Beirut
VUM: Variants under monitoring
VOI: Variants of interest
VOC: Variants of concern
PPE: Personal protective equipment
EMR: Eastern Mediterranean Region
IRB: Institutional Review Board
