# Supplementary material for: Genomic surveillance of SARS-CoV-2 in COVID-19 vaccinated healthcare workers in Lebanon
Source: BMC Med Genomics. 2023 Jan 27;16:14. doi: 10.1186/s12920-023-01443-9 (PMC9880935; doi:10.1186/s12920-023-01443-9)
Supplement: Supplementary file 1 — Additional file 1. Fig. S1: SARS-CoV-2 lineages and variants detected among the sequenced samples. [file 12920_2023_1443_MOESM1_ESM.docx]

**Supplementary figure 1**: SARS-CoV-2 lineages and variants detected among the sequenced samples.
